# Supplementary material for: LTA4H extensively associates with mRNAs and lncRNAs indicative of its novel regulatory targets
Source: PeerJ. 2023 Mar 10;11:e14875. doi: 10.7717/peerj.14875 (PMC10010175; doi:10.7717/peerj.14875)
Supplement: File S2 [file peerj-11-14875-s005.zip › Raw data files/Motif_results/cims/LTA4H_IP_2_vs_LTA4H_input_2/homerResults.html]

.// - Homer de novo Motif Results


# Homer *de novo* Motif Results (.//)

Known Motif Enrichment Results  
Gene Ontology Enrichment Results  
If Homer is having trouble matching a motif to a known motif, try copy/pasting the matrix file into
STAMP  
More information on motif finding results: HOMER
| Description of Results
| Tips
  
Total target sequences = 286516  
Total background sequences = 2582648  
\* - possible false positive  

|  |  |  |  |  |  |  |  |  |
| --- | --- | --- | --- | --- | --- | --- | --- | --- |
| Rank | Motif | P-value | log P-pvalue | % of Targets | % of Background | STD(Bg STD) | Best Match/Details | Motif File |
| 1 |  | 1e-924 | -2.129e+03 | 7.75% | 4.75% | 7.6bp (12.6bp) | hsa-miR-3126-3p MIMAT0015377 Homo sapiens miR-3126-3p Targets (miRBase)(0.659) More Information \| Similar Motifs Found | motif file (matrix) |
| 2 |  | 1e-867 | -1.996e+03 | 21.37% | 16.57% | 9.0bp (15.3bp) | hsa-miR-516a-3p MIMAT0006778 Homo sapiens miR-516a-3p Targets (miRBase)(0.631) More Information \| Similar Motifs Found | motif file (matrix) |
| 3 |  | 1e-543 | -1.252e+03 | 18.15% | 14.56% | 10.1bp (12.9bp) | hsa-miR-1260b MIMAT0015041 Homo sapiens miR-1260b Targets (miRBase)(0.715) More Information \| Similar Motifs Found | motif file (matrix) |
| 4 |  | 1e-468 | -1.079e+03 | 1.62% | 0.71% | 9.7bp (17.0bp) | hsa-miR-4455 MIMAT0018977 Homo sapiens miR-4455 Targets (miRBase)(0.748) More Information \| Similar Motifs Found | motif file (matrix) |
| 5 |  | 1e-446 | -1.028e+03 | 1.89% | 0.91% | 8.7bp (9.6bp) | hsa-miR-4794 MIMAT0019967 Homo sapiens miR-4794 Targets (miRBase)(0.627) More Information \| Similar Motifs Found | motif file (matrix) |
| 6 |  | 1e-376 | -8.675e+02 | 1.53% | 0.72% | 11.0bp (10.1bp) | hsa-miR-4456 MIMAT0018978 Homo sapiens miR-4456 Targets (miRBase)(0.728) More Information \| Similar Motifs Found | motif file (matrix) |
| 7 |  | 1e-318 | -7.322e+02 | 1.49% | 0.75% | 9.0bp (9.7bp) | hsa-miR-5095 MIMAT0020600 Homo sapiens miR-5095 Targets (miRBase)(0.766) More Information \| Similar Motifs Found | motif file (matrix) |
| 8 |  | 1e-294 | -6.788e+02 | 6.41% | 4.78% | 8.5bp (14.5bp) | hsa-miR-495 MIMAT0002817 Homo sapiens miR-495 Targets (miRBase)(0.755) More Information \| Similar Motifs Found | motif file (matrix) |
| 9 |  | 1e-286 | -6.600e+02 | 21.64% | 18.80% | 11.4bp (13.7bp) | hsa-miR-1827 MIMAT0006767 Homo sapiens miR-1827 Targets (miRBase)(0.739) More Information \| Similar Motifs Found | motif file (matrix) |
| 10 |  | 1e-286 | -6.587e+02 | 1.93% | 1.10% | 9.3bp (18.4bp) | hsa-miR-3976 MIMAT0019361 Homo sapiens miR-3976 Targets (miRBase)(0.701) More Information \| Similar Motifs Found | motif file (matrix) |
| 11 |  | 1e-255 | -5.891e+02 | 10.03% | 8.12% | 10.9bp (10.8bp) | hsa-miR-4267 MIMAT0016893 Homo sapiens miR-4267 Targets (miRBase)(0.694) More Information \| Similar Motifs Found | motif file (matrix) |
| 12 |  | 1e-251 | -5.794e+02 | 2.73% | 1.77% | 10.2bp (14.5bp) | hsa-miR-136 MIMAT0000448 Homo sapiens miR-136 Targets (miRBase)(0.704) More Information \| Similar Motifs Found | motif file (matrix) |
| 13 |  | 1e-239 | -5.512e+02 | 1.68% | 0.97% | 9.2bp (10.5bp) | hsa-miR-216a MIMAT0000273 Homo sapiens miR-216a Targets (miRBase)(0.697) More Information \| Similar Motifs Found | motif file (matrix) |
| 14 |  | 1e-172 | -3.976e+02 | 3.54% | 2.60% | 10.0bp (10.3bp) | hsa-miR-4472 MIMAT0018999 Homo sapiens miR-4472 Targets (miRBase)(0.729) More Information \| Similar Motifs Found | motif file (matrix) |
| 15 |  | 1e-158 | -3.650e+02 | 0.82% | 0.43% | 10.1bp (9.8bp) | hsa-miR-4795-3p MIMAT0019969 Homo sapiens miR-4795-3p Targets (miRBase)(0.736) More Information \| Similar Motifs Found | motif file (matrix) |
| 16 |  | 1e-151 | -3.479e+02 | 1.01% | 0.57% | 9.8bp (9.5bp) | hsa-miR-325 MIMAT0000771 Homo sapiens miR-325 Targets (miRBase)(0.688) More Information \| Similar Motifs Found | motif file (matrix) |
| 17 |  | 1e-93 | -2.146e+02 | 0.28% | 0.12% | 10.0bp (11.6bp) | hsa-miR-21\* MIMAT0004494 Homo sapiens miR-21\* Targets (miRBase)(0.658) More Information \| Similar Motifs Found | motif file (matrix) |
| 18 |  | 1e-45 | -1.052e+02 | 9.21% | 8.42% | 11.4bp (14.4bp) | hsa-miR-138-2\* MIMAT0004596 Homo sapiens miR-138-2\* Targets (miRBase)(0.729) More Information \| Similar Motifs Found | motif file (matrix) |
